# Supplementary material for: Examining changes in gramicidin current induced by endocannabinoids
Source: PLoS One. 2025 Aug 18;20(8):e0313903. doi: 10.1371/journal.pone.0313903 (PMC12360604; doi:10.1371/journal.pone.0313903)
Supplement: S1 File — (DOCX) [file pone.0313903.s001.docx]

**SUPPLEMENTARY FIGURES**

**
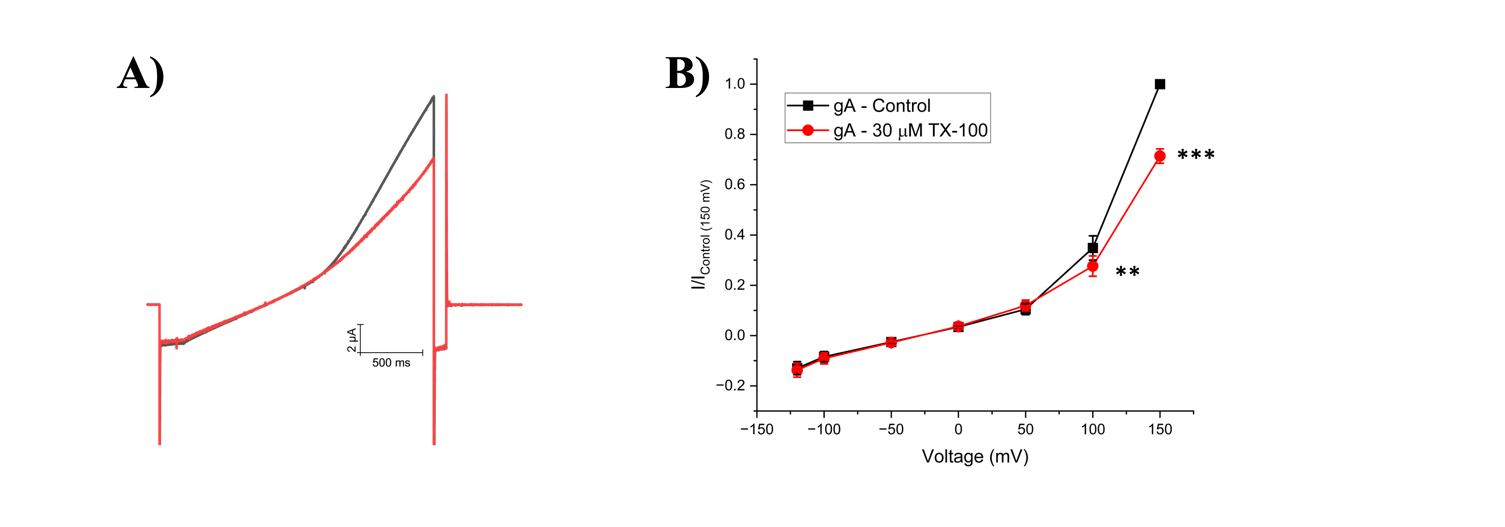
**

**Figure S1:** **Triton-X 100 (TX-100) inhibits cationic gramicidin currents.** **A)** Representative gA current traces in presence of TX-100. **B)** Averaged cationic gramicidin currents of TX-100. Test currents were averaged at -120 mV, -100 mV, -50 mV, 0 mV, +50 mV, +100 mV, and +150 mV. (n = 10; ** = P < 0.01, *** = P < 0.001)

C9

C1

C18

**Figure S2: Structural properties of endocannabinoids.** Gramicidin function was evaluated against three endocannabinoids characteristics: chain length, number of unsaturated bonds and the position of the first unsaturated bond. Representative endocannabinoid: Oleoyl Ethanolamide (OEA), has an 18-carbon tail length, one degree of unsaturation at the 9^th^ carbon position. Carbon count starts at the C1 position located near the ethanolamide head group.

**Table S1. Paired student T test P-values**. Statistics of differences of averaged cationic currents at voltage +150, +100 and -120 mV in the presence of FAE endocannabinoids and TX-100.

| **Endocannabinoid**​ | **Change in current at 150 mV** | **P-value (+150 mV)**​ | **Change in current at 100 mV** | | **P-value (+100 mV)**​ | | **Change in current at -120 mV** | **P-value (-120 mV)**​ |
| --- | --- | --- | --- | --- | --- | --- | --- | --- |
| **AEA​†** | 0.01±0.02 | 0.85 | | 0.03±0.03 | | 0.05 | -0.02±0.02 | 0.08 |
| **αLnEA†​** | 0.13±0.05 | 0.02 | | 0.08±0.04 | | 0.005 | -0.03±0.02 | 0.003 |
| **ArEA** | 0.03±0.05 | 0.58 | | 0.06±0.04 | | 0.1 | -0.03±0.02 | 0.02 |
| **DEA​** | 0.08±0.03 | 0.02 | | 0.05±0.05 | | 0.05 | -0.02±0.02 | 0.11 |
| **LEA​†** | 0.19±0.03 | 0.005 | | 0.09±0.06 | | 0.009 | -0.06±0.04 | 0.01 |
| **NEA​†** | 0.29±0.08 | 0.004 | | 0.09±0.07 | | 0.006 | -0.04±0.04 | 0.01 |
| **OEA​†** | -0.08±0.02 | 0.002 | | -0.014±0.03 | | 0.39 | -0.007±0.03 | 0.52 |
| **oxy-AEA†​** | 0.42±0.08 | 3.90E-04 | | 0.23±0.05 | | 3.12E-04 | -0.05±0.02 | 0.002 |
| **POEA​** | 0.09±0.03 | 0.01 | | 0.05±0.02 | | 0.007 | -0.03±0.02 | 0.009 |
| **SEA​** | 0.13±0.05 | 0.02 | | 0.07±0.04 | | 0.12 | -0.03±0.04 | 0.34 |
| **TEA​** | 0.15±0.05 | 0.02 | | 0.04±0.04 | | 0.16 | -0.01±0.03 | 0.12 |
| **γLnEA​** | 0.05±0.03 | 0.16 | | 0.03±0.03 | | 0.04 | -0.01±0.02 | 0.27 |
| **TX-100†** | -0.38±0.04 | <0.0001 | | -0.09±0.06 | | 0.001 | -0.01±0.03 | 0.29 |
| **DMSO** | 0.04±0.03 | 0.21 | | 0.03±0.04 | | 0.16 | -0.02±0.03 | 0.09 |

**†** representative gramicidin current traces and averaged current-voltage relationship shown in manuscript

**Table S2. Paired student T test P-values**. Statistics of differences of averaged cationic currents at voltage +150, +100 and -120 mV in the presence of 2-MG endocannabinoids.

| **Endocannabinoid**​ | **Change in current at 150 mV** | **P-value (+150 mV)**​ | **Change in current at 100 mV** | | **P-value (+100 mV)**​ | | **Change in current at -120 mV** | **P-value (-120 mV)**​ |
| --- | --- | --- | --- | --- | --- | --- | --- | --- |
| **1-AG** | -0.02±0.01 | 0.08 | | 0.02±0.03 | | 0.14 | -0.01±0.02 | 0.05 |
| **1-MrG​** | 0.03±0.05 | 0.62 | | 0.02±0.06 | | 0.55 | -0.01±0.04 | 0.05 |
| **1-OrG†** | -0.18±0.04 | 0.007 | | -0.05±0.04 | | 0.09 | -0.01±0.02 | 0.17 |
| **1-SG†** | 0.14±0.02 | 0.001 | | 0.04±0.04 | | 0.01 | -0.02±0.03 | 0.09 |
| **1-OG** | -0.009±0.03 | 0.81 | | 0.01±0.03 | | 0.71 | -0.02±0.01 | 0.06 |
| **2-AG†** | -0.02±0.06 | 0.70 | | 0.02±0.03 | | 0.19 | -0.01±0.01 | 0.03 |
| **2-LG** | -0.02±0.04 | 0.58 | | 0.01±0.03 | | 0.59 | -0.02±0.02 | 0.04 |
| **2-PG†** | 0.12±0.04 | 0.01 | | 0.06±0.04 | | 0.009 | -0.03±0.03 | 0.01 |
| **2-SG†** | 0.14±0.05 | 0.02 | | 0.06±0.05 | | 0.04 | -0.02±0.03 | 0.09 |
| **Glycerol** | 0.04±0.03 | 0.22 | | 0.006±0.03 | | 0.58 | 0.01±0.04 | 0.26 |

**†** representative gramicidin current traces and averaged current-voltage relationship shown in manuscript

**Table S3.** Parametric values and statistical significance for linear correlation of FAE endocannabinoids.

| **Structural Property​** | **Intercept** | **Slope​** | **Pearsons’s r** | **R-Square** | **P-value** |
| --- | --- | --- | --- | --- | --- |
| Number of Carbons | 0.94 ± 0.30 | 0.00 ± 0.01 | 0.08 | 0.06 | 0.80 |
| Degree of Unsaturation | 1.02 ± 0.05 | 0.00 ± 0.02 | -0.02 | 0.10 | 0.95 |
| Position of 1^st^ Unsaturated bond | 0.98 ± 0.05 | 0.01 ± 0.01 | 0.29 | 0.08 | 0.37 |
| LogP | 0.94 ± 0.19 | 0.01 ± 0.03 | 0.14 | 0.02 | 0.67 |
| LogD (pH 7.4) | 1.01 ± 0.21 | 0.00 ± 0.04 | 0.01 | 0.00 | 0.98 |

**Table S4.** Parametric values and statistical significance for non-linear (curvilinear) correlation of 2-MG endocannabinoids.

| **Structural Property​** | **Offset (y_0_)** | **Center (x_c_)​** | **Width (w)** | **Amplitude (A)** | **Reduced Chi-Sqr** | **R-Square** | **Residual sum of squares** |
| --- | --- | --- | --- | --- | --- | --- | --- |
| Number of Carbons | 0.84 ± 0.11 | 16.5 ± 0.90 | 2.8 ± 1.23 | 0.27 ± 0.11 | 4.4 | 0.73 | 22.02431 |
| P-values | 1.37E-4 | 2.03E-6 | 0.064 | 0.04 | - | - |  |

**Table S5.** Parametric values and statistical significance for linear correlation of 2-MG endocannabinoids.

| **Structural Property​** | **Intercept** | **Slope​** | **Pearson’s r** | **R-Square** | **P-value** |
| --- | --- | --- | --- | --- | --- |
| Number of Carbons | 0.67 ± 0.01 | 0.02 ± 0.00*** | 0.82 | 0.67 | <0.001 |
| Degree of Unsaturation | 1.08 ± 0.03 | -0.03 ± 0.01* | -0.73 | 0.53 | 0.04 |
| Position of 1^st^ Unsaturated bond | 0.91 ± 0.02 | 0.01 ± 0.00*** | 0.92 | 0.84 | <0.001 |
| LogP (saturated) | 0.72 ± 0.03 | 0.06 ± 0.00*** | 0.99 | 0.98 | <0.001 |
| LogP (unsaturated) | 0.87 ± 0.01 | 0.02 ± 0.01 | 0.99 | 0.97 | 0.06 |
| LogD (pH 7.4) (saturated) | 0.69 ± 0.05 | 0.06 ± 0.01* | 0.98 | 0.96 | 0.003 |
| LogD (pH 7.4) (unsaturated) | 0.87 ± 0.03 | 0.02 ± 0.01 | 0.91 | 0.83 | 0.09 |

* = P < 0.05, *** = P < 0.001
